# Effect of Hydrated Ionic Liquid on Photocycle and Dynamics of Photoactive Yellow Protein

**DOI:** 10.3390/molecules26154554

**Published:** 2021-07-28

**Authors:** Utana Umezaki, Miu Hatakenaka, Kana Onodera, Hiroto Mizutani, Suhyang Kim, Yusuke Nakasone, Masahide Terazima, Yoshifumi Kimura

**Affiliations:** 1 Department of Molecular Chemistry and Biochemistry, Faculty of Science and Engineering, Doshisha University, Kyotanabe 610-0321, Japan; uu1@rice.edu (U.U.); hatakenaka.miu.63m@st.kyoto-u.ac.jp (M.H.); 2 Department of Applied Chemistry, Graduate School of Science and Engineering, Doshisha University, Kyotanabe 610-0321, Japan; kana.rn6@gmail.com (K.O.); hiroto.m-0822@ezweb.ne.jp (H.M.); 3 Department of Chemistry, Graduate School of Science, Kyoto University, Kyoto 606-8502, Japan; ksuhyang95@hikari.kuchem.kyoto-u.ac.jp (S.K.); nakasone@kuchem.kyoto-u.ac.jp (Y.N.); mterazima@kuchem.kyoto-u.ac.jp (M.T.)

**Keywords:** hydrated ionic liquid, photoactive yellow protein, photocycle, transient grating spectroscopy, conformational change

## Abstract

The mechanism by which proteins are solvated in hydrated ionic liquids remains an open question. Herein, the photoexcitation dynamics of photoactive yellow protein dissolved in hydrated choline dihydrogen phosphate (Hy[ch][dhp]) were studied by transient absorption and transient grating spectroscopy. The photocyclic reaction of the protein in Hy[ch][dhp] was similar to that observed in the buffer solution, as confirmed by transient absorption spectroscopy. However, the structural change of the protein during the photocycle in Hy[ch][dhp] was found to be different from that observed in the buffer solution. The known change in the diffusion coefficient of the protein was apparently suppressed in high concentrations of [ch][dhp], plausibly due to stabilization of the secondary structure.

## 1. Introduction

**Figure 1:**
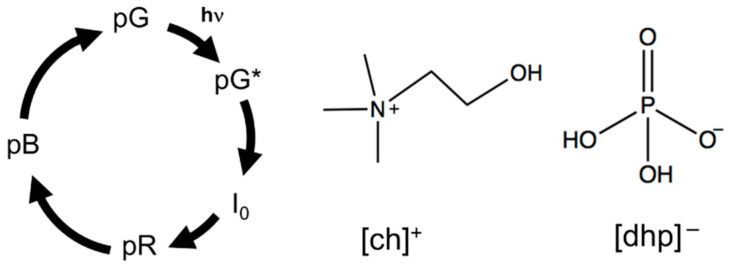
Simplified PYP photocycle model and chemical structure of [ch][dhp].

The interaction between ionic liquids (ILs) and proteins has attracted significant interest. Numerous researchers have studied how proteins are dissolved in ILs and how cations or anions modify the secondary- or higher-order protein structures [1,2,3,4,5,6]. In particular, the role of the alkyl chain length of the IL cations and their relationship with the Hofmeister series have been discussed using various spectroscopic techniques. Although neat ILs can sometimes stabilize and functionalize proteins, it has been revealed that some classes of ILs enhance the stability of proteins when water is added (hydrated ionic liquids, HyILs). Fujita et al. first reported that a mixture of water with choline dihydrogen phosphate ([ch][dhp]) (see Figure 1) enhanced the storage stability of cytochrome C [7,8]. Cytochrome C could be stored in a mixture of water and [ch][dhp] (Hy[ch][dhp]) for more than 18 months without denaturation. They also analyzed the state of dissolved cytochrome C in Hy[ch][dhp] by resonance Raman spectroscopy and FT-IR spectroscopy and found that the structure of the protein did not change in Hy[ch][dhp]. The effect of ILs on protein refolding has been discussed in relation to the stability of the proteins in HyILs [9,10,11,12]. For example, the concentration of guanidine hydrochloride required to denature myoglobin in solution decreased with the addition of 1-butyl-3-methylimidazolium tetrafluoroborate ([Bmim]BF_4_) [10]. On the other hand, ILs with long alkyl chains such as 1-decyl-3-methylimidazolium chloride can induce the refolding of cytochrome C after denaturation by urea and guanidine hydrochloride [11]. Recently, HyILs have been shown to refold protein aggregation [13,14]. Fujita et al. succeeded in refolding recombinant protein aggregates from *Escherichia coli* in Hy[ch][dhp]. Takekiyo et al. reported that heat-aggregated cytochrome C was refolded in a mixture of water and alkylammonium nitrate [13].

In the present study, we demonstrate how the photocycle of a protein is modified by Hy[ch][dhp]. There are numerous photoactive proteins, most of which are activated by conformational changes after photoexcitation. In other words, the conformational change of photoactive proteins can be easily controlled by light irradiation, and monitoring their photoreaction in HyILs will reveal the effect of the HyILs on the protein structure. This study aims to clarify how HyILs affect the conformational change of the photoactive protein by monitoring a photoreaction of a photoactive protein in HyILs. To the best of our knowledge, this is the first study on the photocycle of a protein in HyILs. In the experimental design, we have chosen Hy[ch][dhp] as a typical example of HyILs because it has been wildly tested for protein conservation and refolding, as mentioned in the previous paragraph. As a photoactive protein, photoactive yellow protein (PYP) is utilized [15,16,17,18]. PYP is a relatively small (14 kDa) water-soluble protein and is considered a blue light photoreceptor for a negative phototactic response. The chromophore of PYP is *p*-coumaric acid (4-hydroxycinnamic acid), which is covalently bonded to the side chain of Cys-69 via a thioester linkage [18,19,20]. The photocycle of PYP has been studied under various conditions and for various mutants, and various intermediates and detailed kinetics have been proposed. The main photokinetic may be simplified, as shown in Figure 1 [21]. Ground-state PYP (pG state) absorbs light at approximately 440 nm. Upon photoexcitation, *p*-coumaric acid undergoes photoisomerization, which resulted in a short-lived intermediate state (I_0_) within tens of picoseconds with a red-shifted absorption band. This intermediate is converted to a pR state within a nanosecond, which shows the absorption band around 465 nm [17]. The pR state is then protonated to produce a pB state over several hundreds of microseconds. The pB state is considered as a signaling state, and returns to the original ground state pG within a few seconds [18]. 

The structural change of PYP during the photocycle has been studied in detail using various spectroscopic methods such as transient time-resolved infra-red spectroscopy and X-ray crystallography [22,23,24,25,26]. By applying transient grating (TG) spectroscopy together with the transient absorption spectroscopy, Terazima et al. studied the photochemical process from the pR to pG state in detail. They found that the diffusion coefficient of the pB state (1.00 × 10^−10^ m^2^ s^−1^) is smaller than that of the pG state (1.21 × 10^−10^ m^2^ s^−1^) in buffer solution [27,28]. This difference is ascribed to the conformational change of the N-terminal α-helices in PYP from pR_1_ to pB [29,30]. Additionally, they measured the diffusion coefficients of pG and pB using N-terminal truncated mutants of PYP [29] and reported that truncation of the N-terminal helices led to little change in the diffusion coefficient of pG vs. pB. Considering FT-IR spectroscopic evidence showing the different interactions between the protein and solvent [31], they concluded that the unfolding of the N-terminal α-helix during the photocyclic conversion from pR_2_ to pB induced a change in the interaction between the peptide and solvent, which reduced the diffusivity of PYP compared to that in the folded state. It is quite interesting to determine what happens in the photocyclic reaction of PYP in Hy[ch][dhp]. Herein, the photocyclic reaction of PYP in Hy[ch][dhp] with different concentrations of [ch][dhp] is studied. The circular dichroism (CD) spectrum of PYP in Hy[ch][dhp] is acquired to investigate the ground-state structure before photoexcitation. Furthermore, transient absorption (TA) and TG spectroscopy are applied to monitor the photoinduced reaction dynamics of PYP in Hy[ch][dhp]. 

## 2. Results

### 2.1. CD Spectra of pG State in Hy[ch][dhp]

**Figure 2:**
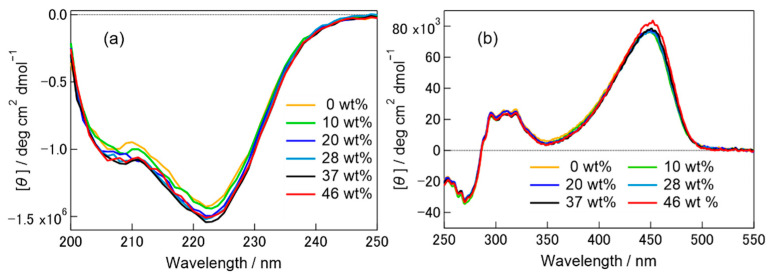
CD spectra of PYP in phosphate buffer (pH 8.0) containing 100 mM NaCl and various concentrations of Hy[ch][dhp] in (**a**) 200–250 nm and (**b**) 250–550 nm. Ellipticity was converted to molar ellipticity per protein.

The absorption profile of the ground state pG did not show any significant change upon addition of [ch][dhp] to the PYP aqueous solution, although the absorption peak shifted to longer wavelength with increasing wt% of [ch][dhp] (see Figure S1, Supplementary Materials). This indicates that the chromophore is not removed from the protein even in solutions with a large wt% of Hy[ch][dhp]. Similar red-shift of the absorption spectrum has been reported for a mutant of less hydrogen-bonding ability with phenolate of the chromophore [32]. Therefore, [ch][dhp] may relax the hydrogen-bonding structure around the chromophore to some extent. Figure 2 shows the CD spectra of PYP in the pG state in solutions with different wt% of Hy[ch][dhp] (the weight percentage of [ch][dhp] in solution) in the (a) far-UV region (200–250 nm) and (b) UV-Vis region (250–550 nm). Since the CD intensities in these two regions are different, they were measured at different PYP concentrations ((a) 5.4 μM and (b) 103 μM). The spectra obtained in the buffer solution both in (a) and (b) were similar to those reported previously [33,34]. As shown in the figure, the CD spectrum changed negligibly with increasing [ch][dhp]-to-water ratio. In Figure 2a, the conformational charge of the backbone protein could be estimated. The intensity of the CD peak at 222 nm, which indicates the existence of the α-helix, did not show a meaningful change with increasing the concentration of [ch][dhp], although there was a small gap observed in the spectra above the 20 wt% of [ch][dhp]. The CD spectra from 250 to 550 nm did not show any change, although the spectrum for the 46 wt% of [ch][dhp] showed some increase in the intensity at 450 nm. The CD bands from 300 to 500 nm mostly arise from the chromophore [34], and it can be said that the secondary structure around the chromophore was not affected by [ch][dhp]. From these observations, we can safely conclude that the structure of the pG state of PYP did not show a meaningful change with the addition of [ch][dhp].

### 2.2. Time Profiles of the Transient Absorption of PYP in Hy[ch][dhp]

It has been reported that the maximum absorption wavelength of PYP changes during the photocycle in buffer solution: pG (λ_max_ = 446 nm), pR (λ_max_ = 465 nm), and pB (λ_max_ = 355 nm) [17]. By monitoring the transient absorption at 436 nm, which is close to the peak maximum of the pG state, the depopulation of the pG state due to conversion to pB via the pR state was analyzed. In the buffer solution, a bleaching signal appeared within a few nanoseconds after illumination with a 460 nm light. Afterward, a further increase in the bleaching intensity was observed due to the change in the absorption wavelength of the pR_2_ vs. pB state on the microsecond time-scale. The TA signal (ΔOD(t)) can be expressed using a bi-exponential function:

(1) $$\Delta \mathrm{OD}\left(t\right)=a_{1}\;\exp\left(-\frac{t}{\tau_{1}}\right)+a_{2}\;\exp\left(-\frac{t}{\tau_{2}}\right),$$

where *τ*_1_ is assigned to the time constant of the transformation from pR_2_ to pB’, in which the N-terminal α-helix unfolds and *τ*_2_ is assigned to the transformation from pB’ to pB. 

The values of *τ*_1_ and *τ*_2_ in buffer solution were reported as 170 μs and 1.0 ms, respectively [27,28]. Simultaneously, upon irradiation with a 460 nm light, in the short time region where the pB’ or the pB state shows an absorption, an increase in the TA signal around 365 nm was observed on the same time-scale. Within the time-scale of milliseconds to seconds, the bleaching was reversed due to the recovery of the pG state (from the pB state). 

**Figure 3:**
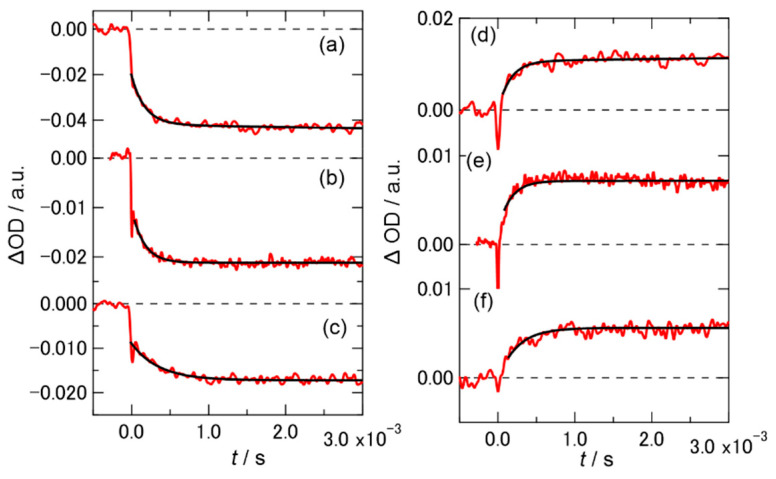
Time profile of the TA at 436 and 365 nm for PYP in (**a**,**d**) 10 wt%, (**b**,**e**) 30 wt%, and (**c**,**f**) 49 wt% Hy[ch][dhp]; (**a**–**c**) 436 nm, (**d**–**f**) 365 nm. Schemes follow the same formatting.

Figure 3 shows the short time profiles of the TA signals of PYP, monitored at 436 nm (a−c) and 365 nm (d−f), with various concentrations of Hy[ch][dhp]. The bleaching signal was observed at 436 nm, and the absorption signal was observed at 365 nm, which indicated that the photocyclic reaction occurred while producing the pB state, as in the buffer solution. The time profiles of PYP in the 10 wt% solution were fitted by a bi-exponential function, and the obtained time constants are listed in Table S1 (Figure S2, Supplementary Materials shows the signal in the long time span of the initial beaching signal at 436 nm). The TA signals of the protein in the 30 and 49 wt% solutions were fitted using a single-exponential function. The time constant for the conversion from pR to pB was not significantly different from that in the buffer solution. Figure S3, Supplementary Materials shows the recovery of the bleaching signal in the long-time region after irradiation at 436 nm. Recovery of the bleaching signal was also observed in the Hy[ch][dhp] solution, which suggested that the photocyclic reaction was completed in Hy[ch][dhp]. The time constants determined from the fitting are listed in Table S1, where the time constant for recovery to the ground state was slower than that observed in the buffer solution.

### 2.3. TG Signals of PYP in Hy[ch][dhp]

**Figure 4:**
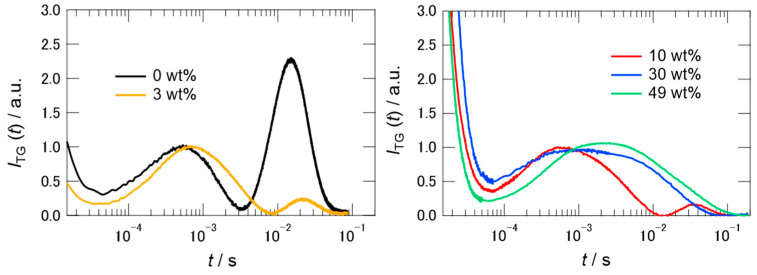
Typical TG signals of PYP in buffer and 3 wt % of [ch][dhp] (left), and in different wt% of [ch][dhp] (right). The values of *q*^2^ are 6.33 × 10^11^ m^−2^ (left), and 5.08 × 10^11^ m^−2^ (10 wt%), 6.60 × 10^11^ m^−2^ (30 wt%), and 7.20 × 10^11^ m^−2^ (49 wt%) (right). The signal intensities were normalized relative to the peak at 10^−3^ s.

Figure 4 shows the TG signals of PYP in different concentrations of Hy[ch][dhp] at similar *q* values, where *q* is the grating wavenumber determined by the wavelength of the pump pulsed light and the incident angle between the pulses. The signal obtained in the buffer solution agrees well with that reported previously [28]. Here, the initial peaks of the thermal grating after photoexcitation are not shown in the figure, where only the tails of the thermal grating signal are shown. Compared with the left figure showing the TG signal in the buffer solution, the signals of PYP in Hy[ch][dhp] were significantly different and dependent on the concentration of [ch][dhp], in contrast to the case of the TA signals. According to a previous study, the first rise and decay signals were assigned to the population dynamics for conversion of pR to pB, and the second rise and decay signals were assigned to the diffusion of pG and pB [28]. The TG signal (*I*_TG_(*t*)) in the buffer solution was simulated using the following equation:

(2) $$I_{\mathrm{TG}}\left(t\right)={\left[A\exp\left(-D_{\mathrm{th}}q^{2}t\right)+B\exp\left(-k_{B}t\right)+C\exp\left(-k_{C}t\right)+D\exp\left(-D_{\mathrm{pG}}q^{2}t\right)+E\exp\left(-D_{\mathrm{pB}}q^{2}t\right)\right]}^{2}$$

where *D*_th_ is the thermal diffusivity; *D*_pB_ and *D*_pG_ are the diffusion coefficients of the pB and pG states, *k_B_* and *k_C_* represent the rate constants of the conversion from pR to pB, which were determined from the transient absorption signal (1/*τ*_1_ and 1/*τ*_2_ in Equation (1)). 

The signs of the coefficients in moving from *A* to *E* were determined by the change in the refractive index for each process, and followed the order: negative, positive, positive, negative, and positive when arranged in time order from fast to slow [28]. Because some components depend on the process of diffusion across the grating (the component including the *q*-dependence), the shape of the signal changed with *q*. As shown in Figure 4, even in 3 wt% [ch][dhp] solution, the second rise and decay in the figure, representing the diffusion of pG and pB, became dramatically smaller. The intensity of the signal decreased further in the 10 wt% solution. In the 30 wt% solution, the second rise and decay signal vanished, and the shape of the first rise and decay signal changed compared with that in the 10 wt% solution. A further increase in the concentration of [ch][dhp] did not dramatically change the signal shape, although the signal decayed slightly slower.

**Figure 5:**
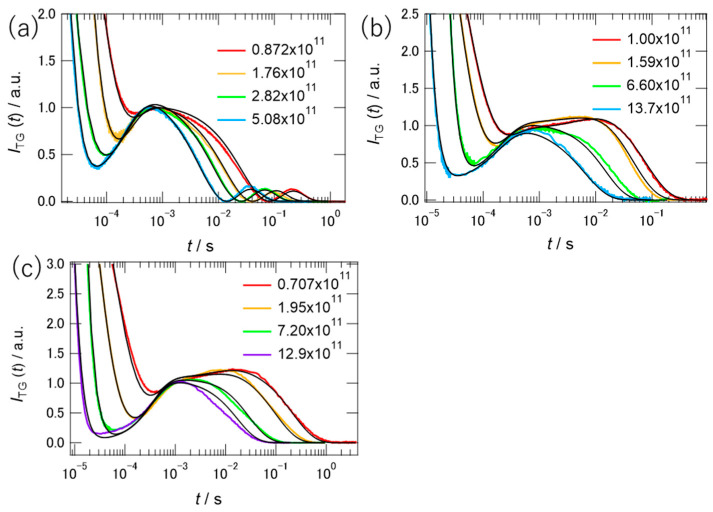
TG signals of PYP in (**a**) 10, (**b**) 30, and (**c**) 49 wt% of [ch][dhp]. The signal intensities were normalized relative to the peak at 10^−3^ s. The values of *q*^2^ in (**a**−**c**) are listed in the graph in the unit of m^−2^. The black curves are fitting curves.

To decompose the signal into individual components, several measurements were performed at different *q*-values. Figure 5 summarizes the TG signals of PYP at different *q* values in different concentrations of [ch][dhp]. As shown in the figure, the shape of the TG signal was dependent on the *q*-value, indicating several contributions to the signal that were not related to the diffusional process. To determine the diffusion coefficients of the pG and pB states, global fitting of the TG signals at different *q* values was performed simultaneously. Equation (2) was used to fit the protein signal obtained in the 10 wt% solution because the signal shape was the same as that in the buffer solution. In the fitting, we used the time constants of *k_B_* and *k_C_* as 1/*τ*_1_ (5750 s^−1^) and 1/*τ*_2_ (180 s^−1^) in Equation (1), where these values were determined by TA measurement. However, the third component *C* in Equation (2) was found to be unnecessary for the fitting, and *C* was assumed to be zero. The signs of the coefficients from *A* to *E* were the same as those in the buffer solution [28]. In the fitting, the relative intensities among pre-exponential coefficients except *A* were fixed for the signals of different *q*-values. The black curves in the figure show the fitting results, which capture almost all signal traces. From the global fitting, the respective values of *D*_pB_ and *D*_pG_ in 10 wt% Hy[ch][dhp] were determined as 8.0(±1.2) × 10^−11^ m^2^ s^−1^ and 9.2(±1.2) × 10^−11^ m^2^ s^−1^. The parameters obtained from the fit are listed in Table S2, Supplementary Materials.

The TG signal shapes were quite different in the 30 and 49 wt% solutions from those in the buffer and 10 wt% solutions. At first, we tried to fit the signals to Equation (2), assuming the similar relative amplitudes of pre-exponential coefficients estimated for the signals of 10 wt% solution. The results of the trail simulations assuming the different diffusion coefficients of the pB and pG states like in the buffer solution are shown by the broken curves in Figure S4, Supplementary Materials. Unless we reduced the relative amplitudes of coefficients *D* and *E* by a factor of 10, we could not simulate the TG signal at any *q*-value. Inspecting various possibilities of different parameter sets, we found that these signals can be simulated by assuming that the diffusion coefficients of pB and pG were very close to each other. Therefore, we assumed that *D*_pB_ and *D*_pG_ are the same. Under this assumption, the TG signal was modeled by the following equation:

(3) $$I_{\mathrm{TG}}\left(t\right)={\left[A\exp\left(-D_{\mathrm{th}}q^{2}t\right)+B\exp\left(-k_{B}t\right)+C\exp\left(-k_{C}t\right)+D\exp\left(-D_{\mathrm{pGpB}}q^{2}t\right)\right]}^{2},$$

where *D*_pGpB_ is the diffusion coefficient of the pG and pB states. If we assume the signs of the coefficients *A* to *D* as negative, positive, positive, and negative, the simulated signals correspond to the black curves in Figure 5b,c.

The simulation works well, although there were some deviations in the intermediate time regions. The rate constant, *k_B_*, was determined by the transient absorption signal at 6500 s^−1^ for the 30 wt% solution and 4000 s^−1^ for the 49 wt% solution. Although the slower time constant was not detected using TA, a component of *k_C_* (140 s^−1^) was required to simulate the signal. The relative amplitudes of pre-exponential coefficients are also shown in Table S2. The value of coefficient *D/B* obtained by the fit to Equation (3) is comparable to the difference between *D/B* and *E/B* (Equation (2)) for the 10 wt% solution, which supports the assumption of the same diffusion coefficients between *D*_pG_ and *D*_pB._ The obtained diffusion coefficients were 6.4(±0.1) × 10^−11^ m^2^ s^−1^ (30 wt%) and 2.6(±0.1) × 10^−11^ m^2^ s^−1^ (49 wt%).

**Figure 6:**
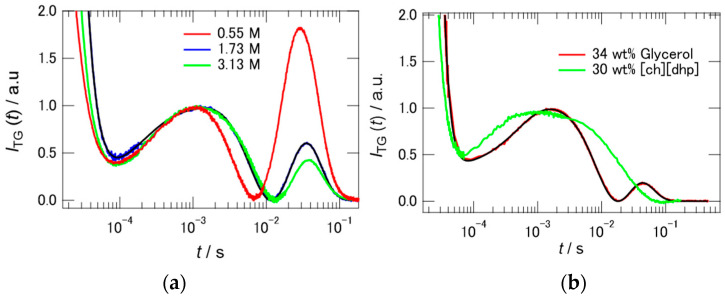
(**a**) TG signals of PYP in corresponding molar concentration of NaCl to 10, 30, 50 wt% [ch][dhp] solution. The *q*^2^ values are 5.49 × 10^11^ (0.55 M), 4.16 × 10^11^ (1.73 M), and 4.92 × 10^11^ (3.13 M) m^−2^. (**b**) Comparison of the TG signals between in the 34 wt% glycerol solution (*q*^2^ = 6.90 × 10^11^ m^−2^) and in the 30 wt% [ch][dhp] solution (*q*^2^ = 6.60 × 10^11^ m^−2^) at the similar *q* values. The signal intensities were normalized relative to the peak at 10^−3^ s both in (**a**,**b**).

Before discussing the reaction dynamics of PYP in Hy[ch][dhp], we check two factors which may affect the photocycle of PYP: one is the ionic strength of the solution, and the other is the viscosity of the solvent. The addition of [ch][dhp] to the buffer solution increases the ionic strength of the solution. Borucki et al. reported that the addition of KCl to a protein solution affects the stability of the intermediate state of the protein [35]. Figure 6a shows the TG signals of PYP in buffer solutions with different concentrations of NaCl. NaCl solutions with concentrations of 0.55, 1.75, and 3.13 M were prepared, where the concentrations of salt correspond to those of 10, 30, and 50 wt% Hy[ch][dhp]. The intensity of the signal due to mass diffusion decreased with increasing salt concentration. However, the signal persisted, even at high salt concentrations (3.13 M), unlike in the case of the Hy[ch][dhp] solution. The TA signal was also acquired in 1.75 M NaCl solution, where it was found that a similar photocyclic reaction occurred, although the rate of conversion from pB to pG was rather fast (see Figure S5 and Table S1, Supplementary Materials). The TG signal in the salt solution (1.75 M) was fitted by Equation (4), where the rate of recovery from pB to pG was substituted into Equation (2), as follows:

(4) $$I_{\mathrm{TG}}\left(t\right)={\left[A\exp\left(-D_{\mathrm{th}}q^{2}t\right)+B\exp\left(-k_{B}t\right)+C\exp\left(-k_{C}t\right)+D\exp\left(-D_{\mathrm{pG}}q^{2}t\right)+E\exp\left(-(D_{\mathrm{pB}}q^{2}+k_{E})t\right)\right]}^{2},$$

where *k_B_* and *k_C_* were determined to be 6200 and 430 s^−1^, respectively, from the TA results. 

The rate of recovery to pG (*k_E_*) was assumed to be 2.1 s^−1^ from the amplitude-weighted lifetimes determined from the TA signal. From analysis of the single *q*-trace, the diffusion coefficients of the pB and pG states in the salt solution were estimated to be 9.1(±0.5) × 10^−11^ m^2^ s^−1^ and 11.0(±0.5) × 10^−11^ m^2^ s^−1^, respectively. 

The addition of [ch][dhp] to the buffer solution also increases the viscosity of the solution. Figure S6, Supplementary Materials shows the plots of the viscosity against the [ch][dhp] concentration. As shown in the figure, the viscosity increases with increasing concentration of [ch][dhp], where the viscosity increase is dramatic with more than 25 wt% of [ch][dhp]. To test the effect of the solvent viscosity, we measured the TG signal in aqueous glycerol solution (34 wt% of glycerol), which shows comparable viscosity (2.45 mPa s) with the 30 wt% Hy[ch][dhp]. Figure 6b shows the TG signals in both solutions. The TG signals are quite different from each other. The TG signal in aqueous glycerol solution shows a signal similar to the one observed in the buffer solution, although the intensity of the second peak is relatively small. The signal shape clearly suggests that the diffusion coefficient of the pB state is different from that of the pG state in the aqueous glycerol solution. The TA signal was also acquired in the aqueous glycerol solution, where it was found that a similar photocyclic reaction occurred, although the rate of conversion from pB to pG was fast (see Figure S7 and Table S1, Supplementary Materials). The reduction of the intensity of the second peak is probably due to the fast recovery time from pB to pG. The TG signal in the aqueous glycerol solution was fitted by Equation (4), as in the case of the salt solution. The values of *k_B_* and *k_C_* were determined to be 2360 and 240 s^−1^, respectively, from the TA results. The rate of recovery to pG (*k_E_*) was assumed to be 6.9 s^−1^ from the amplitude-weighted lifetimes determined from the TA signal. From analysis of the single *q*-trace, the diffusion coefficients of the pG and pB states in the glycerol solution were estimated to be 6.1 × 10^−11^ and 4.4 × 10^−11^ m^2^ s^−1^, respectively.

## 3. Discussion

The TA results indicate that the photocyclic reaction was completed even in the 49 wt% Hy[ch][dhp] solution. In other words, the isomerization of *p*-coumaric acid proceeded as in the buffer solutions. However, the TG signals indicate that the protein dynamics, especially the translational diffusion coefficients, are strongly dependent on the concentration of [ch][dhp]. In the buffer solution, the diffusion coefficients of the pB and pG states differ because of the conformational change of the N-terminal α-helix. The unfolded α-helix undergoes stronger interaction with the solvent molecules, which reduces the diffusion coefficient. On the other hand, in the Hy[ch][dhp] solution at concentrations higher than 30 wt%, we could not simulate the TG signal by using the different diffusion coefficients of the pB and pG states. By assuming that *D*_pG_ and *D*_pB_ are the same, the TG signal could be simulated reasonably. It has been reported that in HyILs, the diffusion coefficient of a protein in its unfolded structure is smaller than that of the protein in its native structure [9,12]. Sasmal et al. reported that the diffusion coefficient of human serum albumin is significantly smaller in the unfolded state than in the native form in 1-methyl-3-pentylimidazolium bromide with water [9]. Pabbathi and Samanta reported that the diffusion coefficient of an unfolded form of cytochrome C is smaller than that of the native form in hydrated ammonium ILs, although the effect of the ionic concentration was negligible [12]. From these observations, the similar diffusion coefficients of the pB and pG states suggests similarity of the conformation of the protein in the two states. According to the CD spectrum shown in Figure 2, the secondary structure of the ground state is not affected by the presence of [ch][dhp]. Therefore, it is reasonable to consider that the α-helix is not unfolded in the pB state and that PYP undergoes a photocyclic reaction with the folded α-helix. As mentioned in the introduction, Hy[ch][dhp] stabilizes the protein. In other words, conformational change may be suppressed by [ch][dhp].

The diffusion coefficients of PYP in Hy[ch][dhp] decreased with increasing concentration of [ch][dhp]. The major contribution to this change is the increasing viscosity of the solution with [ch][dhp] (Figure S6). According to the Stokes–Einstein equation, the diffusion coefficient is related to the solvent viscosity(*η*) by the following equation:

(5) $$D_{i}=\frac{k_{B}T}{6\pi \eta r}$$

where *i* is the chemical species, *k_B_* is the Boltzmann constant, *T* is the absolute temperature, *η* is the viscosity, and *r* is the hydrodynamic radius. 

Although this equation does not necessarily hold due to the complex interaction between the protein and solvent, it may guide the discussion of the viscosity-dependence of the diffusion coefficient. 

**Figure 7:**
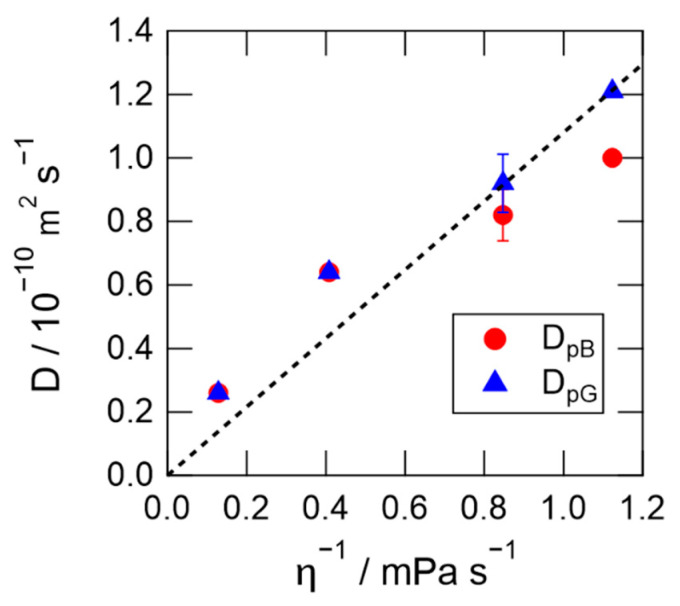
Dependence of diffusion coefficients on the inverse of the viscosity.

Figure 7 shows the dependence of the diffusion coefficients on the inverse of the solvent viscosity. The broken line in the figure represents the linear correlation between *D*_pG_ and *η*^−1^ using the data for the protein in the 0 and 10 wt% Hy[ch][dhp] solutions. The diffusion coefficient clearly increases with increasing *η*^−1^, although the dependence is not strictly linear. The diffusion coefficient is faster at high concentrations of Hy[ch][dhp] (smaller *η*^−1^) than that predicted from the linear relation (broken line) in low-concentration Hy[ch][dhp] (larger *η*^−1^). On the other hand, the diffusion coefficient of the pB state in the high-concentration NaCl solution (1.75 M) was smaller than that of the pG state (Figure 6a). This suggests that the unfolded α-helix interacts more strongly with the solvent, even if numerous ions are dissolved in the solution. It is also to be noted here that the diffusion coefficient of the pB state is different from that of the pG state in the aqueous glycerol solution, which has a similar viscosity to the 30 wt% [ch][dhp] solution. The diffusion coefficient of the pG state is somewhat faster than the prediction from the SE prediction, and gives a similar value to that obtained in the 30 wt% Hy[ch][dhp], while the value of pB is clearly smaller than the value of pG. Therefore, it can be safely said that the viscosity is not the factor which prohibits the conformational change from pG to pB.

Notably, the time for recovery from the pB to pG state in NaCl solution is not very different from that in the buffer solution. The recovery time in the aqueous glycerol solution is faster in buffer solution. On the other hand, the time is much longer in Hy[ch][dhp]. At present, we are not sure of the reason for the difference. It has been reported that the mutation of Y98 by Q dramatically decelerates the recovery time, where Y98 is located near the chromophore [35]. Thus, a subtle environmental change around the chromophore as is demonstrated in the red-shift of the absorption spectrum in Hy[ch][dhp] (Figure S1) may affect the time-scale of the photocycle. 

## 4. Materials and Methods

Choline dihydrogen phosphate ([ch][dhp]) was synthesized following a previously reported procedure [8]. Briefly, choline chloride (extra pure; Nacalai Tesque Inc., Kyoto, Japan) and potassium hydroxide (>85%; Sigma-Aldrich Co. LLC, St. Louis, MO, USA) were dissolved in 2-propanol and potassium chloride was precipitated by freezing overnight. The supernatant was removed under reduced pressure. The compound was dissolved in water and converted to [ch][dhp] by adding phosphoric acid (>85%; Nacalai Tesque). The water was removed under reduced pressure, and the product was further purified by recrystallization with methanol/acetone. The purity of [ch][dhp] was checked by ^1^H-NMR and Mohr’s method. H-purity was >99.5% and the chloride content was <0.1%. ^1^H-NMR was measured by a JNM-ECA300W (JEOL Ltd., Tokyo, Japan).

PYP was prepared as previously reported [28]. PYP samples in 10, 30, and 49 wt% Hy[ch][dhp] for the TG measurements were prepared by mixing [ch][dhp] and PYP samples in 10 mM Tris-HCl with 100 mM NaCl buffer solution (Tris-HCl NaCl; pH = 8.0). To acquire the CD spectrum using a circular dichroism spectrometer (J-720WI; JASCO Corp., Tokyo, Japan), PYP solutions of Hy[ch][dhp] in phosphate buffer (pH 8.0) containing 100 mM NaCl were prepared. For these solutions, the concentration of [ch][dhp] was confirmed from the relative intensity of the ^1^H-NMR peak of water vs. that of the cholinium cation. PYP solutions with high salt (NaCl) concentrations comparable to those of Hy[ch][dhp] were prepared similarly. The glycerol solution (34 wt% of glycerol) of similar viscosity to the 30 wt% Hy[ch][dhp] was prepared by mixing glycerol with the buffer solution. The viscosity of Hy[ch][dhp], NaCl solutions, and aqueous glycerol solution was determined using a cone-plate-type viscometer (DV-II pro; Brookfield Engineering Laboratories, Inc., Middleboro, MA, USA). 

The experimental setup for the transient grating (TG) measurement is described elsewhere [36]. Briefly, an output pulse (460 nm) of a dye laser (ND6000; Continuum, Milpitas CA, USA) pumped by a third-harmonic pulse (355 nm) of an Nd:YAG laser (Surelite; Continuum) was used as the excitation pulse. A He-Ne laser (LGK7651-8, 633 nm; Lasos Lasertechnik GmbH, Jena, Germany) was used as the probe beam. The TG signal was detected using a photomultiplier, and the signal was transferred to an oscilloscope (Waverunner 44Xi; Lecroy, Chestnut Ridge, NY, USA). The value of the grating lattice vector was determined by the thermal grating signal decay rate of bromocresol purple in methanol, measured with the same optical geometry, using the values of the thermal diffusivity of methanol. For the transient absorption measurements at 364 and 436 nm, an Hg lamp was used as the light source, and the wavelength was separated by a proper optical band-pass filter. The changes in the optical density with and without the pump pulse (ΔOD) were measured at each probe wavelength. The TA spectrum (ΔOD(*λ*, *t*)) was determined using the following equation:

(6) $$\Delta \mathrm{OD}\left(\lambda ,t\right)=-\log\left(\frac{I\left(\lambda ,t\right)}{I_{0}\left(\lambda ,t\right)}\right)$$

where $I_{0}\left(\lambda ,t\right)$ is the initial probe light intensity without the pump pulse and I(λ,t) is the probe light intensity after passing through the sample, which was excited by the pump pulse.

## 5. Conclusions

In the present study, the photocycle of PYP in Hy[ch][dhp] was revealed. Although the photoreaction of the chromophore proceeds as in buffer solution, the protein did not undergo conformational changes in the presence of [ch][dhp]. The present results suggest that [ch][dhp] suppresses the structural changes in proteins, which are closely related to the stability of the proteins in Hy[ch][dhp]. Further studies on the dynamics of the folding or unfolding of proteins are desirable to elucidate the interaction between HyILs and proteins.

## Data Availability

The data presented in this study are available on request from the corresponding author.

## Supplementary Materials

The following are available online, Figure S1: UV-Vis absorption spectra of PYP in different wt % solution of Hy[ch][dhp], Figure S2: time profiles of the bleaching of the transient absorption of PYP in the extended time range, Figure S3: time profiles of the bleaching recovery of the transient absorption of PYP in the long time range, Figure S4: trial fits of the TG signal in the 30 wt% [ch][dhp] solution using Equation (2), Figure S5: time profile of the transient absorption at 436 nm for PYP in 1.75 M NaCl solution, Figure S6: viscosity of Hy[ch][dhp] vs. concentration of [ch][dhp], Figure S7: time profile of the transient absorption at 436 nm for PYP in 34 wt% glycerol solution, Table S1: time constants obtained from the transient absorption analysis, Table S2: Parameters obtained and used for the global-fit of the TG signals.

## Sample Availability

Samples of the compounds are not available from the authors.
