# Supplementary material for: Effect of Hydrated Ionic Liquid on Photocycle and Dynamics of Photoactive Yellow Protein
Source: Molecules. 2021 Jul 28;26(15):4554. doi: 10.3390/molecules26154554 (PMC8348629; doi:10.3390/molecules26154554)
Supplement: Supplementary file 1 [file molecules-26-04554-s001.zip › molecules-1256509-supplementary.pdf]

molecules

MDPI

Supplementary Materials

Effect of Hydrated Ionic Liquid on Photocycle and Dynamics of Photoactive Yellow Protein

Utana Umezaki [1], Miu Hatakenaka [1], Kana Onodera [2], Hiroto Mizutani [2], Suhyang Kim [3], Yusuke Nakasone [3], Masahide Terazima [3] and Yoshifumi Kimura [1,2,*]

[1] Department of Molecular Chemistry and Biochemistry, Faculty of Science and Engineering, Doshisha University, Kyotanabe, 610-0321, Kyoto, Japan; uu1@rice.edu (U.U.); hatakenaka.miu.63m@st.kyoto-u.ac.jp (M.H.)
[2] Department of Applied Chemistry, Graduate School of Science and Engineering, Doshisha University, Kyotanabe, 610-0321, Kyoto, Japan; kana.rn6@gmail.com (K.O.); hiroto.m-0822@ezweb.ne.jp (H.M.)
[3] Department of Chemistry, Graduate School of Science, Kyoto University, Kyoto 606-8502, Kyoto, Japan; ksuhyang95@hikari.kuchem.kyoto-u.ac.jp (S.K.); nakasone@kuchem.kyoto-u.ac.jp (Y.N.); mterazima@kuchem.kyoto-u.ac.jp (M.T.)
* Correspondence: yokimura@mail.doshisha.ac.jp

Abstract: The mechanism by which proteins are solvated in hydrated ionic liquids remains an open question. Herein, the photoexcitation dynamics of photoactive yellow protein dissolved in hydrated choline dihydrogen phosphate (Hy[ch][dhp]) were studied by transient absorption and transient grating spectroscopy. The photocyclic reaction of the protein in Hy[ch][dhp] was similar to that observed in the buffer solution, as confirmed by transient absorption spectroscopy. However, the structural change of the protein during the photocycle in Hy[ch][dhp] was found to be different from that observed in the buffer solution. The known change in the diffusion coefficient of the protein was apparently suppressed in high concentrations of [ch][dhp], plausibly due to stabilization of the secondary structure.

Keywords: hydrated ionic liquid; photoactive yellow protein; photocycle; transient grating spectroscopy; conformational change

Table S1. Time constants of the reaction obtained by the transient absorption spectrum analysis. The value in the parenthesis is the relative amplitude to that of the slower component (a_1/a_2 in eq. (1) at 436 nm).

	pR → pB	pB → pG
buffer	170 s (0.5) , 1 ms [a)]	150 ms (13), 2s [b)]
10 wt% [ch][dhp]	170 s (5.2), 5.7 ms	2.7 s
30 wt% [ch][dhp]	160 s	2.3 s
49 wt% [ch][dhp]	250 s	1.3 s
1.75 M NaCl solution	160 s (0.53), 1.3 ms	0.26 s (1.7), 0.85 s
34 wt% glycerol solution	420 s (0.42), 4.1 ms	70 ms (8.1), 0.83 s

a) Refs. 21 and 22. b) Ref. 17.

Table S2. Relative amplitudes of pre-exponential coefficients to the value *B* and time constants obtained by the global fit for the TG signals. k_B and k_C are determined by the transient absorption signals and are kept constant for the global fitting. The pre-exponential factor *A* was excluded from the global fitting considering the probable contribution of the multi-photon excitation which increased the thermal grating signal (factor *A*).

wt%	Eq.	D_{th} / 10^{-7} m² s⁻¹	k_B / μs⁻¹	*C/B*	k_C /μs⁻¹	*D/B*	D_{pG} (or D_{pBpG}) / 10^{-11} m² s⁻¹	*E/B*	D_{pB} / 10^{-11} m² s⁻¹
10	(2)	1.43	5750	0	180	-17.4	9.2	16.0	8.0
30	(3)	1.30	6250	0.357	140	-2.13	6.4		
49	(3)	1.04	4000	0.124	140	-1.16	2.4		

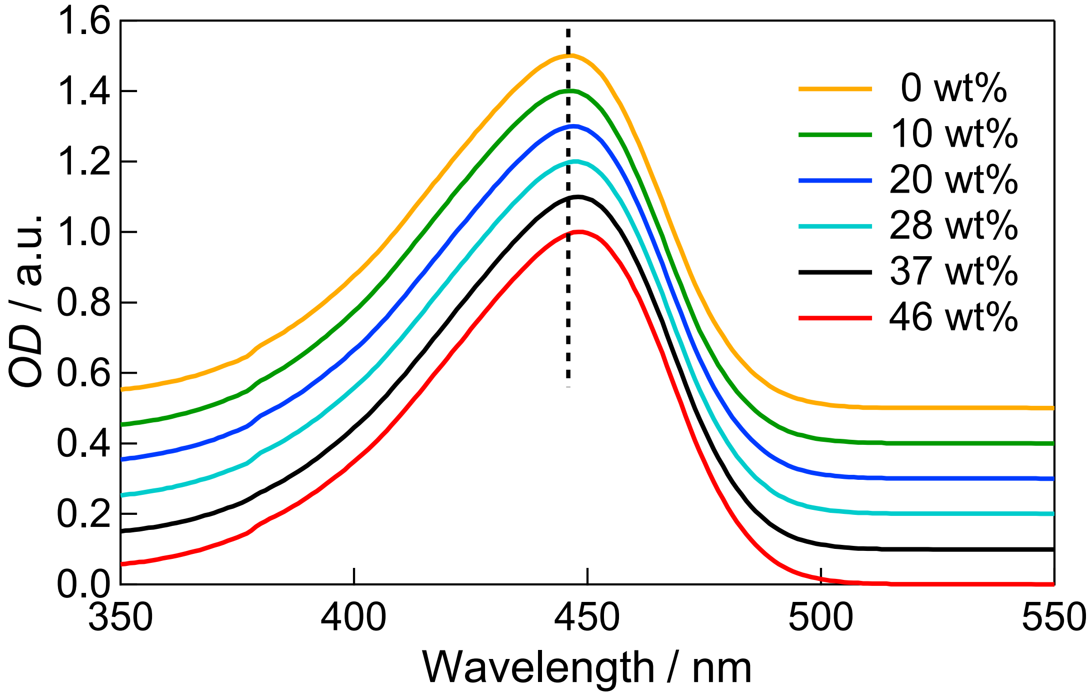

Figure S1. Absorption spectra of PYP in different wt% of Hy[ch][dhp]. Each spectrum is shifted vertically for clarity. The absorbance is normalized at the peak position.

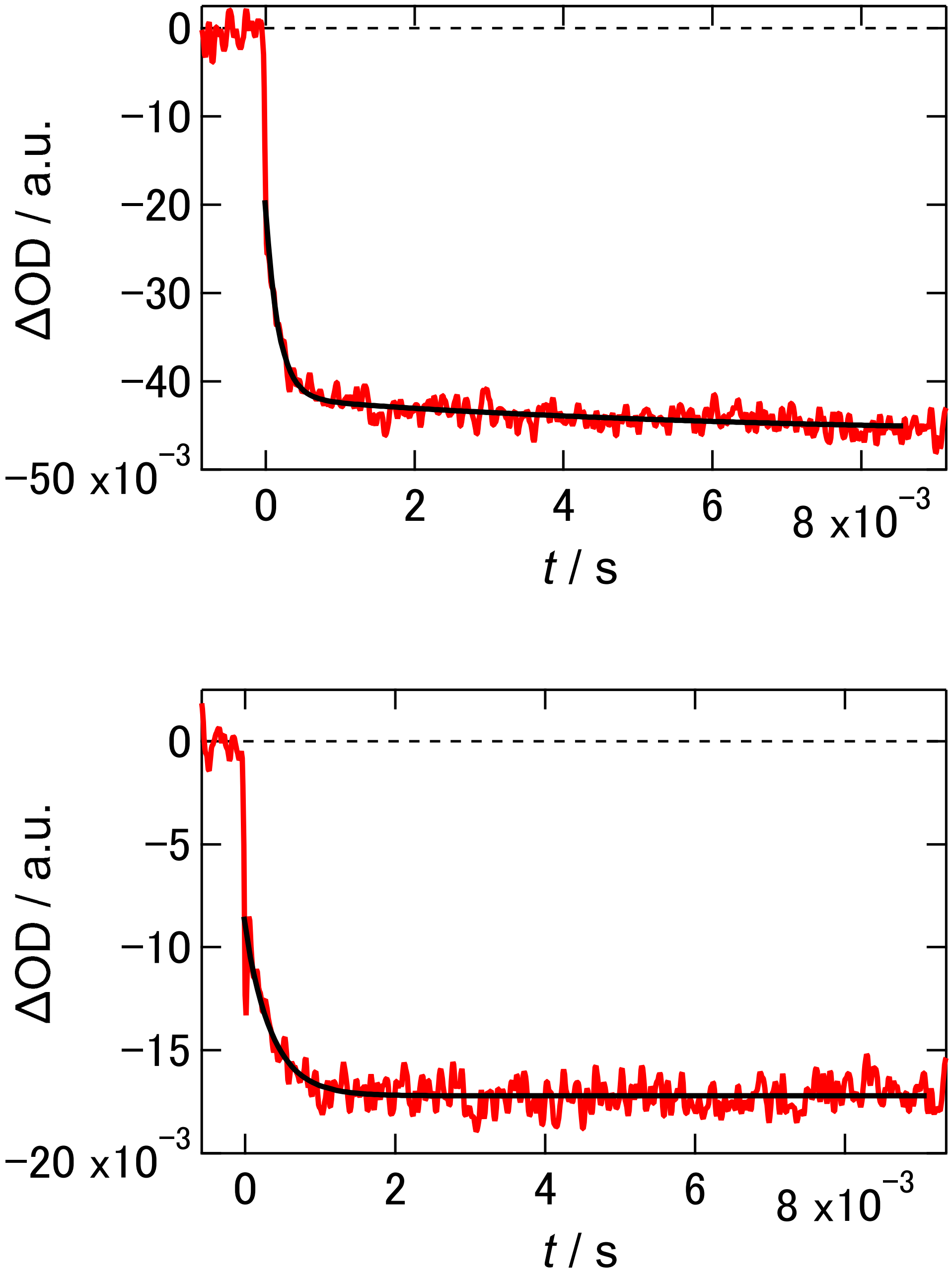

Figure S2. Time profile of the transient absorption at 436 nm for PYP in (upper) 10 wt% and (lower) 50wt% of Hy[ch][dhp].

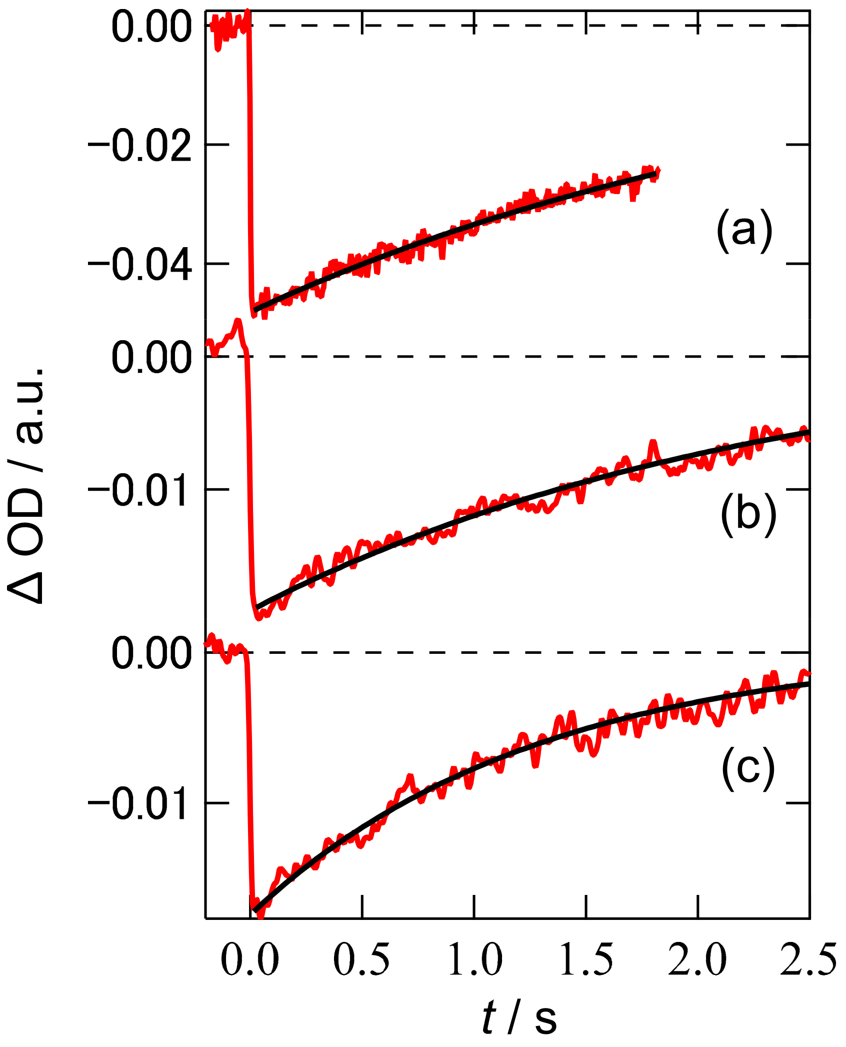

Figure S3. Time profile of the transient absorption at 436 nm in the long time range for PYP in (a) 10 wt%, (b) 30 wt%, and (c) 49 wt% of Hy[ch][dhp.

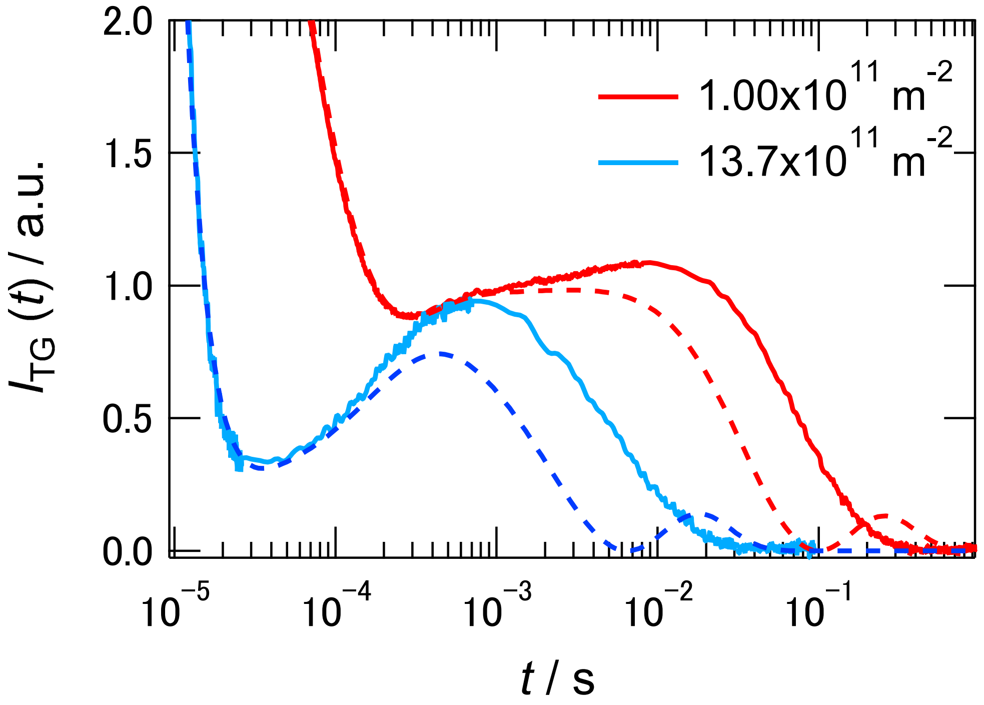

Figure S4. Trial fit (broken curves) to Eq. (2) using plausible parameters for the TG signals in 30 wt% [ch][dhp] solution at different q^2 values (indicated in the figure). The values of D_{pG} and D_{pB} are assumed to be 6.9×10^{-11} m^2 s^{-1} and 5.9×10^{-11} m^2 s^{-1}. The relative amplitudes of the coefficients (*D*/*B* and *E*/*B*) are assumed to be similar to those for 10 wt% [ch][dhp].

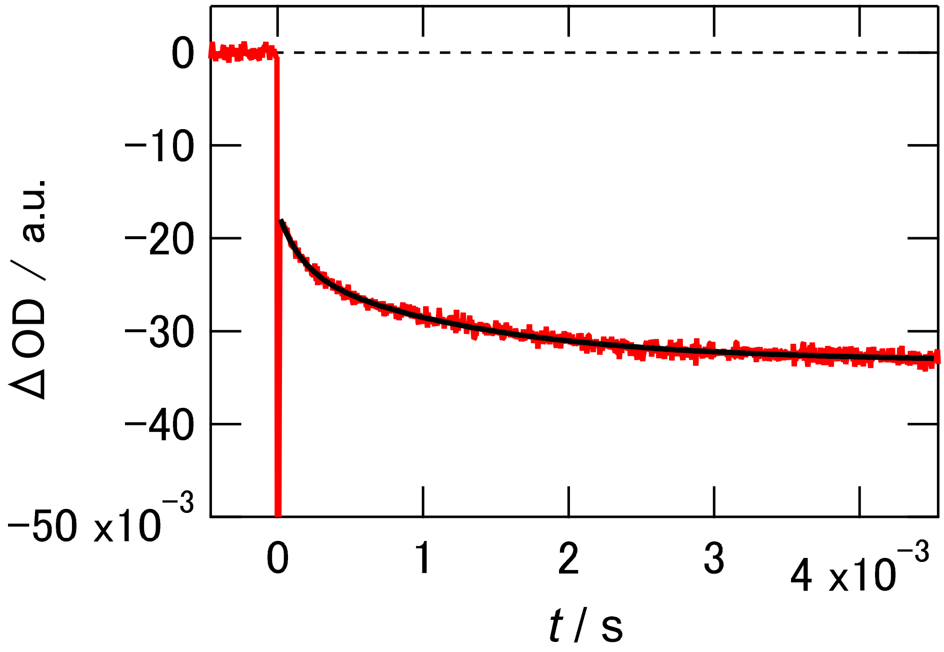

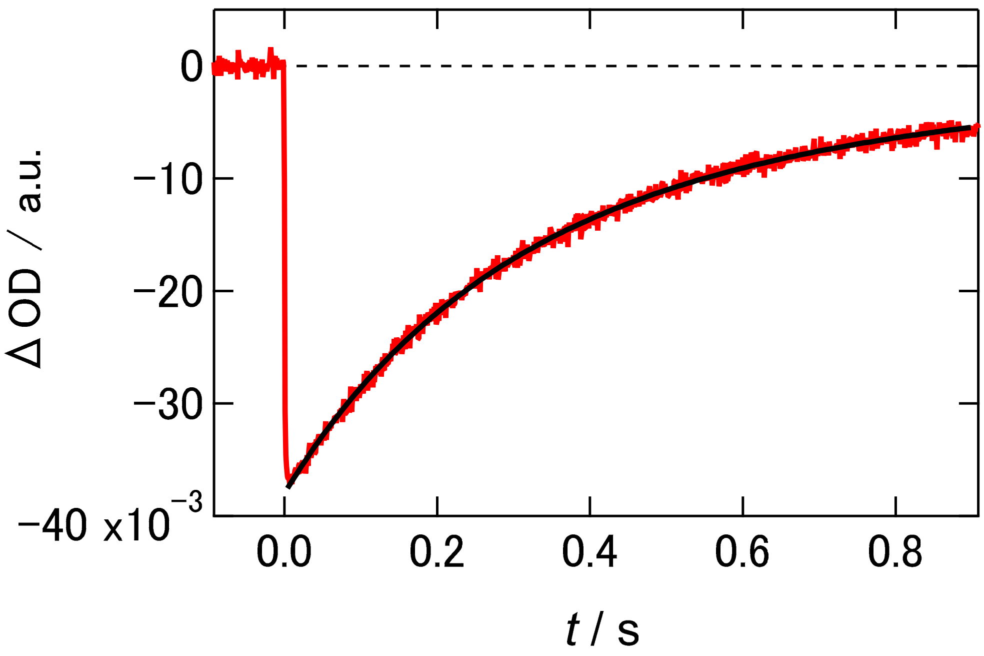

Figure S5. Time profile of the transient absorption at 436 nm for PYP in 1.75 M NaCl solution (upper) in the short time scale and (lower) in the long time scale.

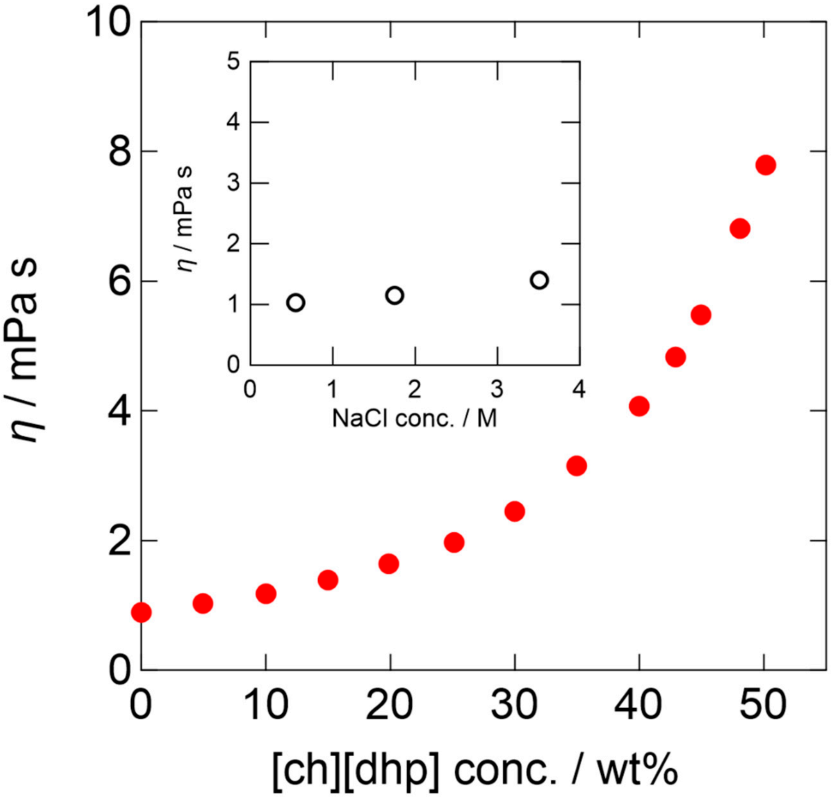

Figure S6. Dependence of viscosity (η) on the wt% of [ch][dhp]. The inset figure shows the dependence of viscosity of the NaCl solution on the NaCl concentration.

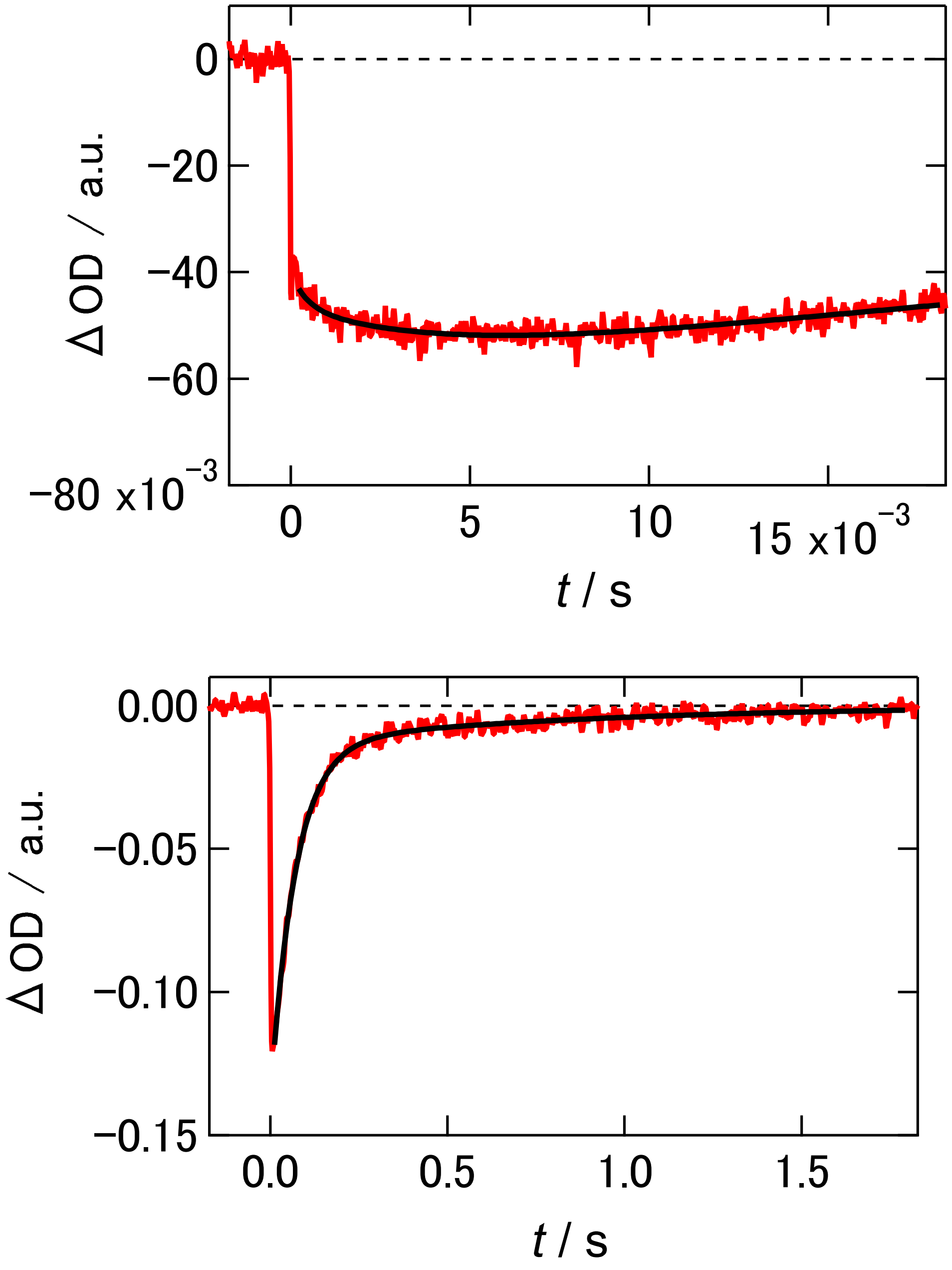

Figure S7. Time profile of the transient absorption at 436 nm for PYP in the 34 wt% glycerol solution (upper) in the short time scale and (lower) in the long time scale.
